# Hypertension in Obese Type 2 Diabetes Patients is Associated with Increases in Insulin Resistance and IL-6 Cytokine Levels: Potential Targets for an Efficient Preventive Intervention

**DOI:** 10.3390/ijerph110403586

**Published:** 2014-03-28

**Authors:** Ljiljana Lukic, Nebojsa M. Lalic, Natasa Rajkovic, Aleksandra Jotic, Katarina Lalic, Tanja Milicic, Jelena P. Seferovic, Marija Macesic, Jelena Stanarcic Gajovic

**Affiliations:** Clinic for Endocrinology, Diabetes and Metabolic Diseases, Clinical Center of Serbia, Faculty of Medicine, University of Belgrade, Dr Subotica 13, 11000 Belgrade, Serbia; E-Mails: ljlukic@eunet.rs (L.L.); nrajkovic@open.telekom.rs (N.R.); aleksandra.z.jotic@gmail.com (A.J.); katarina.s.lalic@gmail.com (K.L.); icataca@gmail.com (T.M.); jpseferovic@gmail.com (J.P.S.); macesicmarija@gmail.com (M.M.); stanarcicjelena@gmail.com (J.S.G.)

**Keywords:** type 2 diabetes, hypertension, obesity, insulin resistance, IL-6, prevention

## Abstract

Increased body weight as well as type 2 diabetes (T2D) are found to be associated with increased incidence of hypertension, although the mechanisms facilitating hypertension in T2D or nondiabetic individuals are not clear. Therefore, in this study we compared the levels of insulin resistance (IR:OGIS), plasma insulin (PI:RIA) levels, and pro-inflammatory cytokines (IL-6 and TNF-α: ELISA), being risk factors previously found to be associated with hypertension, in T2D patients showing increased body weight (obese and overweight, BMI ≥ 25 kg/m^2^) with hypertension (group A, N = 30), or without hypertension (group B, N = 30), and in nonobese (BMI < 25 kg/m^2^), normotensive controls (group C, N = 15). We found that OGIS index was the lowest (A: 267 ± 35.42 *vs.* B: 342.89 ± 32.0, *p* < 0.01) and PI levels were the highest (A: 31.05 ± 8.24 *vs.* B: 17.23 ± 3.23, *p* < 0.01) in group A. In addition, IL-6 levels were higher in group A (A: 15.46 ± 5.15 *vs.* B: 11.77 ± 6.09; *p* < 0.05) while there was no difference in TNF-α levels. Our results have shown that appearance of hypertension in T2D patients with increased body weight was dependent on further increase in IR which was associated with the rise in pro-inflammatory IL-6 cytokine. The results imply that lifestyle intervention aimed to decrease IR might be beneficial in reducing the risk for hypertension in those T2D individuals.

## 1. Introduction

It has been shown that more than 80% of patients with type 2 diabetes (T2D) will become hypertensive and it has been postulated that both T2D and hypertension represent potent risk factors for the development of different forms of ischemic cardiovascular disorders [1]. However, the relationship between these important risk factors in the pathogenesis of cardiovascular disease (CVD), as well as the possibilities of the modulation of their influences, has not yet been clarified. The mechanisms underlying pathogenesis of CVD in T2D patients with hypertension are found to involve numerous factors but recent evidences have suggested that activation of low-grade inflammation might be a possible trigger of this process [2].

On the other hand, obesity has been identified as a facilitating factor for the development of both T2D and hypertension. In addition, adipose tissue is now recognized as an endocrine organ that is a strong amplifier of insulin resistance (IR) in humans [3]. Interleukin-6 (IL-6) and tumor necrosis factor-alpha (TNF-α) are cytokines with metabolic and/or weight-regulating effects. The role IL-6 plays in obesity and IR remains controversial even after many years of research. Circulating levels of IL-6 are increased in obesity [3,4], and it has been proposed that IL-6 contributes to the pathogenesis of IR in different disease states [5]. The major source of systemic IL-6 is adipose tissue, and reducing fat mass in obesity reduces circulating IL-6 levels [6,7,8]. In addition, IL-6 was found to impair insulin sensitivity, to increase leptin production and lipolysis and to decrease lipoprotein lipase activity in adipocytes [7,8,9].

Many studies have shown that both T2D and hypertension are strongly associated with increased IR and obesity, besides being powerful risk factors for CVD. However, the role of IR and the involvement of low-grade inflammation in the development of hypertension in the settings of already existing T2D and obesity have not yet been elucidated [10,11,12,13,14,15].

In this study, we undertook the analysis of the role of IR and pro-inflammatory cytokines in the development of hypertension in T2D patients with increased body weight (obese and overweight, body mass index, BMI ≥ 25 kg/m^2^). This analysis has revealed the association between the increases in IR and IL-6 and the presence of hypertension in obese and overweight T2D, which suggests that preventive (e.g., lifestyle) intervention improving insulin sensitivity in these individuals might be very important in preventing hypertension in this subset of high-risk individuals.

## 2. Methods

### 2.1. Subjects

We performed a cross-sectional study of 75 subjects: (a) T2D with increased body weight (obese and overweight, BMI ≥ 25 kg/m^2^) and hypertension (group A, n = 30), (b) T2D patients with increased body weight (obese and overweight, BMI ≥ 25 kg/m^2^) without hypertension (group B, n = 30) and nonobese (BMI ≥ 25 kg/m^2^) healthy controls (group C, n = 15). Exclusion criteria were BMI ≥ 35 kg/m^2^, clinically significant renal or hepatic disease, anemia, diabetic retinopathy or symptomatic neuropathy, cardiac failure (New York Heart Association grades III and IV), angina pectoris, or recent myocardial infarction and severe uncontrolled hypertension. T2D was diagnosed in accordance with the criteria of World Health Organization: fasting plasma glucose ≥ 7 mmol or 2 h plasma glucose ≥ 11.1 mmol/L [16]. T2D patients were treated with oral antidiabetic agents, none of them were treated with insulin.

Hypertension was defined as (systolic/diastolic blood pressure (BP) (≥140/≥90 mmHg), according to Seventh Report of the Joint National Committee on Prevention, Detection, Evaluation and Treatment of High Blood Pressure (JNC-7) criteria [17] or currently receiving antihypertensive agents. The patients without hypertension involved in this study were not on any medications lowering BP and were noted to have BP of less than 140/90.

The study was approved by the ethics review committee of Faculty of Medicine, University of Belgrade, Serbia and written informed consent was obtained from each participant. The study was conducted at the Clinic for Endocrinology, Diabetes and Metabolic Diseases, Clinical Center of Serbia, Faculty of Medicine, University of Belgrade.

### 2.2. Study Design

At screening visit at the outpatients clinic subjects were interviewed about medical conditions, current medication and smoking habits. Antihyperglycemic, hypolipidemic and antihypertensive agents were stopped 24–48 h before the metabolic testing.

The presence of obesity was determined by using BMI which was calculated as weight/height^2^ (kg/m^2^). Height was recorded to the nearest 0.5 cm, and weight was measured to the nearest 0.1 kg.

The BP was measured by using a standard mercury sphygmomanometer after the subjects had rested at least 10 min, in three separate measurements in sitting position, by the same doctor. SBP was recorded at the appearance of sounds, and DBP was recorded at the disappearance of sounds (V-phase Korotkov).

In each patient we performed the detection of: (a) insulin sensitivity, (b) plasma insulin (PI), (c) plasma glucose (c) IL-6 and TNF pro-inflammatory cytokines, (c) HbA1c and (d) lipid subfraction levels (total, HDL, LDL cholesterol and triglycerides).

### 2.3. Evaluation of Insulin Sensitivity

Insulin sensitivity was evaluated by using the OGIS method [18]. Briefly, the plasma glucose and PI values measured from the samples during the standard OGTT were used to calculate the OGIS index according to Mari *et al*. [18]. The standard 75-g OGTT was performed after a 12-h overnight fast. Glucose solution was ingested within 2 min, and venous blood samples were collected for measurements of plasma glucose and insulin concentrations at 0, 30, 60, 90, and 120 min after glucose loading [16,18].

### 2.4. Laboratory Measurements

All analyses were carried out during the same day and blood samples drawn by the same study nurse after a 12 h overnight fast and were stored at −70 °C until assayed. PI levels were determined by radioimmunoassay (double antibody kits, INEP, Zemun, Serbia). Plasma glucose concentrations were measured using the glucose oxidase method using Beckman Glucose Analyzer (Beckman Instruments, Fullerton, CA, USA). Glycosylated hemoglobin (HbA1c) levels were determinate using turbidimetric immunoassay for HbA1c (Boehringer Mannheim, Mannheim, Germany). Total cholesterol, HDL cholesterol and triglyceride concentrations were determined with enzymatic methods (Boehringer Mannheim). LDL cholesterol concentrations were calculated using Friedewald formula. TNF-α and IL-6 were measured by ELISA system (ALPCO, Salem, NH, USA).

### 2.5. Statistical Analysis

Data are expressed as means ± SD. Normality of distribution of the data was tested by the Kolmogorov-Smirnov Test, a *p* value greater than 0.05 indicated that the observed distribution of a variable is not statistically different from the normal distribution. Comparison of metabolic variables was done by Kruskal-Wallis Test when the data were not normally distributed. The continuous variables were analyzed with analysis of variance (ANOVA). Data with a *p* value less than or equal to 0.05 were considered statistically significant. The software package SPSS version 16.0 for Windows (Chicago, IL, USA) was used for all computations.

## 3. Results and Discussion

### 3.1. Results

#### 3.1.1. Patients Characteristics

The clinical and metabolic characteristics of the patients and subjects involved in the study are shown at Table 1. No significant differences were seen among groups with respect to mean age, BMI, HbA1c, total cholesterol, triglycerides, and fasting plasma glucose, between the groups of diabetic patients. The HDL cholesterol was significantly lower in group A.

**Table 1:** Clinical and laboratory characteristics in T2D patients with increased body weight and healthy subjects.

	Group			
	A T2D + HTA+	B T2D + HTA−	C Control	*p* value (Group A *vs.* B)
n (M/F)	30 (16/14)	30 (15/15)	15 (7/8)	NS
Age (years)	56.47 ± 3.91	57.67 ± 3.96	44.06 ± 4.51	NS
Duration of diabetes (years)	4.59 ± 1.53	4.44 ± 1.26	-	NS
BMI (kg/m^2^) *	31.20 ± 2.53	30.92 ± 2.34	22.77 ± 2.9	NS
SBP (mmHg) *	140.17 ± 12.25	141.66 ± 13.13	122.03 ± 6.97	NS
DBP (mmHg) *	86.67 ± 9.12	85.56 ± 8.81	78.67 ± 4.81	NS
HbA1c (%) *	6.51 ± 0.66	6.53 ± 0.60	4.71 ± 0.32	NS
FPG (mmol/L) *	7.36 ± 1.40	7.46 ± 1.51	4.06 ± 0.71	NS
Total Ch (mmol/L) *	6.16 ± 0.93	6.14 ± 0.82	5.6 ± 0.98	NS
Triglycerides (mmol/L) *	2.66 ± 1.15	2.37 ± 0.80	1.29 ± 0.59	NS
HDL-Ch (mmol/L) *	0.98 ± 0.11	1.12 ± 0.18	1.58 ± 0.47	*p* < 0.05
LDL-Ch (mmol/L) *	3.85 ± 0.78	3.99 ± 0.75	3.53 ± 0.64	NS
Smoking (n, %)	10 (33.3)	11 (36.3)	5 (33.3)	NS

Data are n, means ± SEM. * *p* ≤ 0.05 A, B *versus* C. T2D: Type 2 Diabetes; HTA: Hypertension; BMI: Body Mass Index; SBP: Systolic Blood Pressure; DBP: Diastolic Blood Pressure; HbA1c: glycosylated hemoglobin; FPG: Fasting Plasma Glucose; Total Ch: Total Cholesterol; HDL-Ch: High Density Lipoprotein Cholesterol; LDL-Ch: Low Density Lipoprotein Cholesterol.

**Figure 1:**
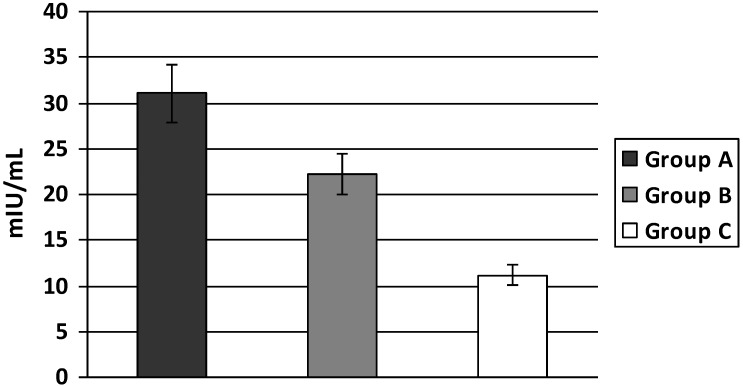
Plasma insulin levels in T2D patients with increased body weight.

Values are expressed as mean ± SE. Bar graph show the value of Plasma Insulin (PI). PI were significantly higher in type 2 diabetic (T2D) patients with increased body weight (obese and overweight) and hypertension compared to T2D patients with increased body weight without hypertension and same relationship were found in comparison to healthy subjects (A *vs.* B *p* < 0.05 and A, B *vs.* C *p* < 0.01).

#### 3.1.2. Insulin Sensitivity

**Figure 2:**
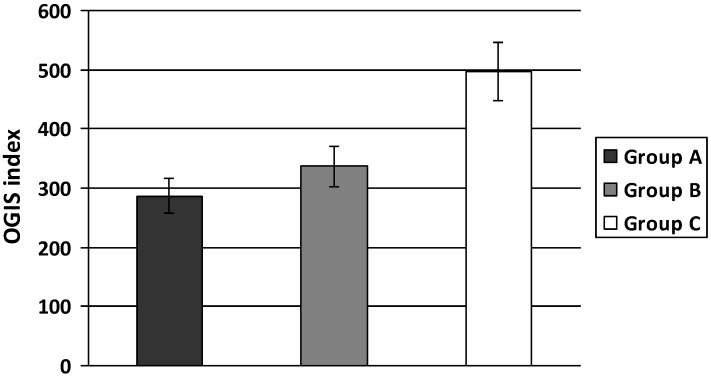
OGIS index in T2D patients with increased body weight.

PI levels were significantly higher in individuals with increased body weight (group A and B) when compared to normal controls and were also higher in obese and overweight individuals with increased body weight and hypertension (group A) *versus* those without hypertension (group B) (A: 31.05 ± 8.24; B: 17.23 ± 3.23; C: 11.12 ± 2.69 mIU/mL, A *vs.* B *p* < 0.05 and A, B *vs.* C *p* < 0.01) (Figure 1). Insulin sensitivity evaluated by OGIS index was significantly lower both in groups A and B compared to group C. In addition, this index was significantly lower in group A compared to group B (A: 267 ± 35.42; B: 342.89 ± 32.0; C: 496.80 ± 63.35 A *vs.* B *p* < 0.01 and A, B *vs.* C *p* < 0.01) (Figure 2).

Values are expressed as mean ± SE. Bar graph show the value of OGIS index. The OGIS index as measurement of peripheral IR was significantly lower in T2D patients with increased body weight and hypertension than in T2D patients with increased body weight with optimal BP and the same relationship were faound in comparison to healthy subjects (A *vs.* B *p* < 0.01 and A, B *vs.* C *p* < 0.01).

#### 3.1.3. Pro-inflammatory Cytokines

**Figure 3:**
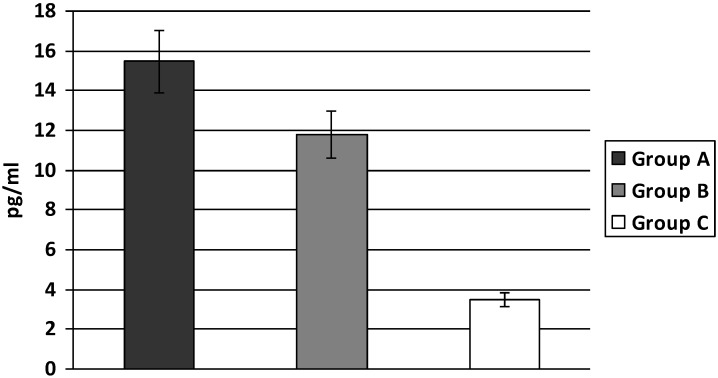
Levels of IL-6 in T2D patients with increased body weight.

IL-6 levels were significantly higher both in group A and B compared to group C. The highest level of IL-6 was found in group A, being significantly higher than in group B (A: 15.46 ± 5.15; B: 11.77 ± 6.09; C: 3.48 ± 1.48 pg/mL, A *vs.* B *p* < 0.05; A, B *vs.* C *p* < 0.01) (Figure 3).

**Figure 4:**
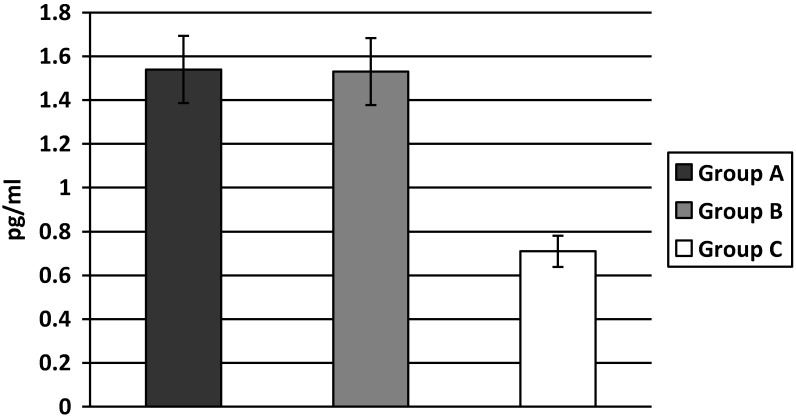
Levels of TNF-α in T2D patients with increased body weight.

The TNF-α levels were higher both in groups A and B when compared to group C, but there was no difference between groups A and B (A: 1.54 ± 0.41; B: 1.53 ± 0.42; C: 0.71 ± 0.30 pg/mL, A *vs.* B *p* = NS; A, B *vs.* C *p* < 0.01) (Figure 4).

Values are expressed as mean ± SE. Bar graph show the value of Interleukin 6 (IL-6). IL-6 were significantly higher in T2D patients with increased body weight and hypertension compared to T2D patients with increased body weight with optimal BP and the same relationship were found in comparison to healthy subjects (A *vs.* B *p* < 0.05; A, B *vs.* C *p* < 0.01).

Values are expressed as mean ± SE. Bar graph show the value of Tumor Necrosis Factor alpha (TNF-α). TNF-α were significantly higher in both groups of T2D patients with increased body weight in comparison to control subjects, there was no difference in TNF-α levels between the groups of T2D patients differing in the presence of hypertension (A *vs.* B *p* = NS; A, B *vs.* C *p* < 0.01).

### 3.2. Discussion

In this study we have found the highest IR (evaluated both by OGIS index and PI levels) in T2D patients with increased body weight (obese and overweight) and hypertension which is strongly associated with increases in the levels of pro-inflammatory cytokines, predominantly IL-6.

IR is thought to be an important pathogenic mechanism in the pathogenesis of essential hypertension [19,20,21,22]. A number of studies have been entirely consistent in showing that lower insulin sensitivity is associated with higher BP [19,20,21,22,23,24].

It was previously demonstrated that insulin sensitivity indices based on the OGTT significantly correlated with the glucose disposal rate (M-value) measured by the glucose clamp technique [18,25]. Previous studies have suggested that OGIS index, calculated from OGTT may provide a reliable estimate of insulin sensitivity especially in patients with T2D [18,25,26,27,28,29]. This was the reason why we used this index of insulin sensitivity based on the 75-g OGTT.

We found that insulin sensitivity is already substantially decreased in T2D patients with increased body weight. Moreover, the OGIS index as measurement of peripheral IR was significantly lower in the obese and overweight T2D patients with hypertension than in those with optimal BP. Our results imply that increases of IR might be an important factor influencing the development of hypertension in obese T2D patients.

Over the past decades many studies have suggested that low-grade inflammation related to obesity might be the key regulator in pathogenesis of T2D [30,31,32,33,34,35,36,37,38,39,40]. It has been confirmed that enlargement of adipose tissue is associated with increases of number of adipose tissue macrophages, which are responsible for increases in plasma concentration of pro-inflammatory cytokines, especially IL-6 and TNF-α expression [31]. IL-6 is released from macrophages of adipose tissue as well as from adipocytes and skeletal muscle [32,33]. *In vitro* and *in vivo* work has shown that IL-6 gene expression and circulating levels of IL-6 may be regulated by insulin and correlate well with central obesity [31,32,33]. TNF-α level is associated with IR, and this cytokine promotes serine phosphorylation of insulin receptor substrate 1 that impairs insulin signaling, resulting IR [33,34,35,36]. These pro-inflammatory cytokines appear in early stage of T2D and they are found to be capable to increase IR directly in adipocytes, muscle and hepatic cells leading to augmentation of the systemic IR [37,38,39].

Our results have confirmed these findings of increased levels of IL-6 and TNF-α in T2D patients with increased body weight (obese and overweight), but among them IL-6 and TNF-α were found to be significantly higher in the hypertensive patients. In addition, in our study the changes in IL-6 showed the same pattern as the increases in IR in the hypertensive obese and overweight T2D individuals.

However, we could not find the significant difference in the levels of TNF-α between hypertensive and normotensive T2D patients with increased body weight. The differences in the pattern of changes between IL-6 and TNF-α in our study might be caused by a different relationship between IR and each of the pro-inflammatory cytokines [5,34,35,36].

In past several decades different epidemiological studies showed the presence of a co-clustering of inflammation and hypertension in patients at high CVD risk [40,41,42,43]. Interestingly, these authors also observed that the association between the low-grade inflammation and the risk of becoming hypertensive remained statistically significant even after adjustment for features of the metabolic syndrome [40]. In addition, some recent epidemiological studies showed that the presence of a low-grade inflammation could anticipate the future development of hypertension [41,42]. This novel observation suggests that the increase in plasma levels of pro-inflammatory cytokines observed among hypertensive patients cannot be solely attributed to the vascular damage induced by high blood pressure [43].

Another factor that might be involved in the pathogenesis of hypertension in the settings of obesity-associated IR is increased sympathetic activity [22]. It has been recognized that obesity represents a condition of increased sympathetic activity, increase in norepinephrine (NE) concentrations and NE renal spillover, and this hyperactivity is associated with tissue IR. In pathogenesis of hypertension, some recent studies emphasize the role of arterial stiffening preceding the development of hypertension [44]. Interestingly, the impairments in pulse wave velocity, a measure of large vessels distension ability, was recently found to be associated with the increases in circulating levels of IL-6 and TNF- α [22,45,46] suggesting that low-grade inflammation may contribute to arterial stiffness.

New lines of research are now investigating the possibility of a direct pathogenic effect of pro-inflammatory mediators in altering mechanisms of vascular tone regulation leading to the onset of high blood pressure [47,48,49], which might clarify the mechanisms linking hypertension and low grade inflammation.

Lifestyle modification, physical activity and nutritional interventions [48,49], may reduce development of diabetes, but also the level of blood pressure and inflammation in patients with hypertension and T2D, which is important for the prevention of cardiovascular diseases [50]. Our results imply that this effect might be achieved by targeting IR and low-grade inflammation, predominantly IL-6 levels. The results of the National programme of early detection and prevention of T2D in Serbia is based on lifestyle modification aiming to reduce the risk not only for T2D but also to its complications and comorbidities, especially hypertension [51]. Our results imply that beneficial effect in that direction might be achieved primarily by targeting IR and low-grade inflammation, predominantly IL-6 levels.

## 4. Conclusions

In conclusion, we found that in obese patients with T2D the development of hypertension depends on the increases in IR and pro-inflammatory cytokines, especially IL-6 levels. Our results imply that lifestyle intervention aimed to decrease IR and chronic inflammation might be beneficial in reducing the risk for hypertension in obese T2D individuals.

## Abbreviations

OGIS: Oral Glucose Insulin Sensitivity index
IL-6: Interleukin-6
TNF-α: Tumor Necrosis Factor-α
BMI: Body Mass Index
